# Exosome-mediated bidirectional immune dysregulation in tuberculosis: proteomic profiling reveals strain-specific strategies of virulent H37Rv and attenuated H37Ra

**DOI:** 10.3389/fimmu.2025.1696299

**Published:** 2026-01-23

**Authors:** Xiuli Zhang, Wenxia Ma, Yuzhu Zheng, Lingna Lyu

**Affiliations:** 1 Department of Gastroenterology and Hepatology, Laboratory for Clinical Medicine, Beijing You’an Hospital Affiliated to Capital Medical University, Beijing, China; 2 Department of Rheumatology and Clinical Immunology, Peking University First Hospital, Beijing, China; 3 Beijing Chest Hospital Affiliated to Capital Medical University, Beijing, China

**Keywords:** exosomes, immune evasion, macrophage, mycobacterium tuberculosis, proteomics, strain virulence

## Abstract

**Introduction:**

Tuberculosis (TB), caused by *Mycobacterium tuberculosis* (*Mtb*), remains a global health crisis, with drug resistance and immune evasion complicating control efforts. *Mtb* subverts macrophage function to establish persistent infection, but the role of exosomes in immune regulation remains poorly understood.

**Methods:**

This study employed iTRAQ-based proteomics to dissect strain-specific immune modulation strategies of virulent H37Rv (RV) and attenuated H37Ra (RA) through macrophage and exosome profiling.

**Results:**

We revealed distinct survival strategies of *Mtb* in Macrophages: RV maintained host cell viability and intracellular proliferation, while RA induced apoptosis. Human proteomic profiling identified significantly more upregulated host proteins in RA-infected macrophages than in RV-infected cells, with RA robustly activating antigen presentation pathways. Conversely, exosomes from infected macrophages exhibited overall protein downregulation, particularly for RV. Strikingly, 24 of the top 25 enriched pathways were upregulated intracellularly but downregulated in exosomes, indicating bidirectional immune dysregulation. Bacterial proteomics revealed that functional proteins were preferentially sorted into exosomes. RV-exosomes were enriched in dormancy regulators (e.g., DevS) and immunosuppressive effectors, while RA-exosomes carried immunogenic antigens leading to robust cytokines releasing such as THF-a, IL-1a and IL-6.

**Discussion:**

Conclusively, Mtb exploits exosomes as “virulence vectors” to deliver RhoGDI and death signals (e.g., Caspse-9), paralyzing systemic immunity while optimizing intracellular survival. Virulence-specific cargo sorting informs novel diagnostics and therapies against TB. However, given the limitations of the in vitro model, future research should incorporate in vivo models and clinical trials to validate these findings.

## 1 Introduction

Tuberculosis (TB), caused by *Mycobacterium tuberculosis* (*Mtb*), remains a global health emergency despite decades of research, prevention and control efforts. According to the latest WHO report, TB accounted for 10.8 million incident cases and 1.25 million deaths in 2022, returning to being the world’s leading cause of death from a single infectious agent (1). Alarmingly, the global TB incidence rate declined by only 8.3% annually between 2015-2023, far below the 2025 target of a 50% reduction set by the End TB Strategy (1). In China, the third largest TB burden country, 741,000 new cases were reported in 2023, with 29,000 cases of multidrug-resistant TB (MDR-TB) and a treatment success rate of 60% (1, 2). These persistent challenges underscore the urgent need for demonstrating the mechanism of TB pathogenesis.

Macrophages, the primary host cells for *Mtb*, play a dual role in the immunopathological interplay between pathogen and host defenses. On one hand, they initiate antimicrobial responses through phagocytosis, reactive oxygen species (ROS) production, and pro-inflammatory cytokine secretion (3, 4). On the other hand, *Mtb* subverts host defenses by inhibiting phagosome-lysosome fusion and inducing necrotic cell death to promote bacterial dissemination (5). Recent studies highlighted strain-specific differences in macrophage responses: virulent H37Rv induces necroptosis to evade immune detection, while attenuated H37Ra triggers apoptosis and robust antigen presentation (6–8).

Exosomes, nanoscale extracellular vesicles (30–150 nm), have emerged as critical mediators of *Mtb*-host interactions. Infected macrophages release exosomes containing bacterial antigens (e.g., 19 kDa lipoprotein) and host-derived immune modulators, which activate dendritic cells and T cell responses (9, 10). Notably, exosome-mediated communication is a conserved mechanism across diverse infectious agents, facilitating viral dissemination in HIV infection and modulating host immune responses in leishmaniasis (11, 12). Proteomic studies have identified exosomal cargoes involved in inflammation and apoptosis (13, 14). However, the differential contribution of exosomes from H37Rv- or H37Ra-infected macrophages to immune regulation remains poorly understood.

To address this knowledge gap, we employed iTRAQ-based proteomics to compare the protein profiles of macrophages and exosomes following infection with H37Rv and H37Ra. Our study aims to: 1) identify strain-specific immune signatures in host cells and exosomes; 2) characterize the role of exosomes in mediating inter-cellular communication during TB infection; and 3) discover potential novel biomarkers for TB diagnosis and therapeutic targets. These findings will provide critical translational evidence to advance host-directed therapies for improved clinical management of *Mtb* infections.

## 2 Materials and methods

### 2.1 Cell culture and infection model

Human monocytic THP-1 cells (ATCC TIB-202) were maintained in RPMI-1640 medium (Gibco 11875093) supplemented with 10% fetal bovine serum (FBS, Gibco 10099141C), 1% penicillin-streptomycin (Gibco 15140122), and 50 μM β-mercaptoethanol (Gibco 21985023), with passaging every 3 days at a 1:5 ratio. For macrophage differentiation, cells were seeded at 1×10^6^ cells/mL and treated with 100 nM phorbol 12-myristate 13-acetate (PMA, Sigma-Aldrich P8139) for 48 hours. Mycobacterium tuberculosis H37Rv (ATCC 27294) and H37Ra (ATCC 25177) were cultured in Middlebrook 7H9 broth (BD 271310) containing 10% OADC enrichment (BD 212351), 0.05% Tween 80 (Sigma-Aldrich P1754), and 0.2% glycerol (Sigma-Aldrich G5516) to mid-log phase (OD_600_=0.6-0.8). Bacterial clumps were dispersed by sonication (30% amplitude, 10 cycles), and viable counts were determined by plating on 7H10 agar (BD 271320). Differentiated macrophages (1×10^6^ cells/well in 6-well plates) were infected with *Mtb* at a multiplicity of infection (MOI) of 10:1 for 4 hours at 37 °C. Extracellular bacteria were eliminated by washing with PBS and treating with 50 μg/mL gentamicin (Gibco 15750060) for 1 hour. Cells were then washed thrice with PBS and cultured in exosome-free medium (RPMI-1640 with 10% exosome-depleted FBS prepared by 100,000g ultracentrifugation for 16 hours). Exosome-depleted FBS was used throughout the post-infection culture period.

### 2.2 Macrophage viability and *Mtb* survival detection

CCK-8 assay for macrophage viability: At designated time points post-infection (8, 24, 48, 72 h post infection), add CCK - 8 reagent (10 μL per 100 μL culture medium) to macrophages cultured in 96-well plates. Incubate for 4 h at 37 °C. Directly measure the absorbance at 450 nm using a microplate reader.

CFU assay for bacterial survival: Lyse infected macrophages at identical time points (24, 48, 72, 96 h post infection) using 0.1% Triton X-100 in PBS for 10 min at room temperature (RT). Serially dilute lysates in PBS with 0.05% Tween 80. Plate dilutions on Middlebrook 7H11 agar with 10% OADC. Incubate 3–4 weeks at 37 °C, then enumerate colonies.

### 2.3 Exosome isolation and characterization

Culture supernatants collected 24 hours post-infection were processed using differential ultracentrifugation: 300g for 10 minutes to remove cells, 2,000g for 20 minutes to pellet debris, 10,000g for 30 minutes to eliminate apoptotic bodies, and finally 100,000g for 70 minutes (Beckman Coulter Optima XPN-100, SW 41 Ti rotor) to isolate exosomes. Exosome pellets were resuspended in PBS and stored at -80 °C. Transmission electron microscopy (TEM) was employed to visualize exosomes. 10 µL of exosome suspension was applied to a 200-mesh formvar/carbon-coated copper grid (Ted Pella, Inc.) and allowed to adsorb for 10 minutes at room temperature (RT). The grid was then negatively stained with 2% aqueous phosphotungstic acid (pH 7.0) for 2 minutes, briefly rinsed with distilled water, and air-dried. Excess liquid was carefully blotted with filter paper between each step. Images were acquired using a JEM-1400 TEM (JEOL, Japan) operating at 80 kV. Western blot was performed to detect exosomal surface marker CD9 and TSG101 (EXOAB-CD9A-1 and EXOAB-TSG101-1, SBI, CA, USA), and an endoplasmic reticulum marker protein, Calnexin (negative control) was detected with anti-Calnexin antibody (ab22595, Abcam, MA, USA). The size distribution of exosomes was measured through NanoSight Tracking Analysis (NTA, Malvern, UK) equipped with a 488 nm laser. Samples were diluted in filtered PBS to achieve an optimal particle concentration of 20–100 particles per frame. The camera level was set to 14, and the detection threshold was set to 5. Five videos of 60 seconds each were captured for every sample. Data were processed with NTA software (version 3.4) to determine the mean and mode particle size and concentration. Immunogold labeling (15) was used to visualize exosomes. Exosome pellets were dropped onto 200 mesh formvar carbon-coated nickel grids, incubated with 50 mM glycine for 15 minutes to quench autofluorescence, followed by blocking with 5% bovine serum albumin (BSA) in PBS for 30 minutes. Grids were then incubated overnight at 4 °C with primary rabbit anti-human antibodies diluted in 1% BSA against CD9 (1:50, EXOAB-CD9A-1, SBI), CD63 (1:50, EXOAB-CD63A-1, SBI), and Hsp70 (1:100, EXOAB-Hsp70A-1, SBI). An antibody against the endoplasmic reticulum marker Calreticulin (1:100, ab2907, Abcam) served as a negative control. After washing five times with 0.1% BSA, grids were incubated for 2 hours at RT with a 10 nm Protein A-gold conjugate (1:50 in 1% BSA/PBS). Grids were washed with PBS and distilled water, negatively stained with 2% phosphotungstic acid, and imaged as described above.

### 2.4 Cytokines detection

Differentiated THP-1 macrophages were treated with the extracted exosomes at ratio (exosome:cell) of 100:1, using MILLIPLEX^®^ milliplex assay (HCYTOMAG-60K-08, Millipore, MA, USA), and analyzed using Luminex 200 platform.

### 2.5 Proteomic analysis

Macrophages were lysed in RIPA buffer (Thermo Fisher 89900) containing protease inhibitors (Roche 04693159001), while exosomes were lysed in 0.1% Triton X-100 (Sigma-Aldrich T8787). Protein concentration was determined using the BCA assay (Thermo Fisher 23227). For iTRAQ labeling, 100 μg protein was reduced with 10 mM DTT at 56 °C for 30 minutes, alkylated with 55 mM iodoacetamide in darkness for 20 minutes, and digested with trypsin (Promega V5111) at a 1:50 ratio overnight. Peptides were labeled with iTRAQ 8-plex reagents (AB Sciex 4392028) according to the manufacturer’s protocol.

Labeled peptides were fractionated by high-pH reverse-phase HPLC (Waters XBridge C18 column, 3.5 μm, 4.6×250 mm) and analyzed on a TripleTOF 6600 mass spectrometer (AB Sciex) operating in data-dependent acquisition mode (350–1250 m/z, 25 MS/MS scans per cycle). Raw data were processed with ProteinPilot 5.0 (Paragon algorithm) against UniProt Homo sapiens (2024_03) for human proteins, and Proteome Discoverer 2.3 (SEQUEST HT) against UniProt *Mycobacterium tuberculosis* (2024_03) for *Mtb* proteins. Quality control included FDR ≤0.01 via Percolator and retention of proteins identified by ≥2 unique peptides.

### 2.6 Statistical analysis

Data are presented as mean ± standard deviation (SD). Group differences were analyzed using Student’s t-test or one-way ANOVA with Tukey’s *post-hoc* test (GraphPad Prism 9). Proteomic data were analyzed in Perseus, with differentially expressed proteins (DEPs) defined as |fold change| ≥1.2 and adjusted p ≤0.05 (Benjamini-Hochberg correction). The DEPs were analyzed and compared using R packages such as VennDiagram, Pheatmap, and ggplot2 were used to draw Venn diagrams, heatmaps, and clustering diagrams.

For human proteins, the DEPs were enriched for differential functions and differential pathways using Ingenuity Pathway Analysis software (IPA) based on z-scores; the interactions between DEPs were also analyzed and functionally classified (Ingenuity^®^ Systems, http://www.ingenuity.com).

For bacterial proteins, COG clustering analysis was performed; functional protein was also analyzed including virulence proteins, antigenic proteins, and membrane-associated proteins.

## 3 Results

### 3.1 Macrophage infection model and exosome characterization

**Figure 1:**
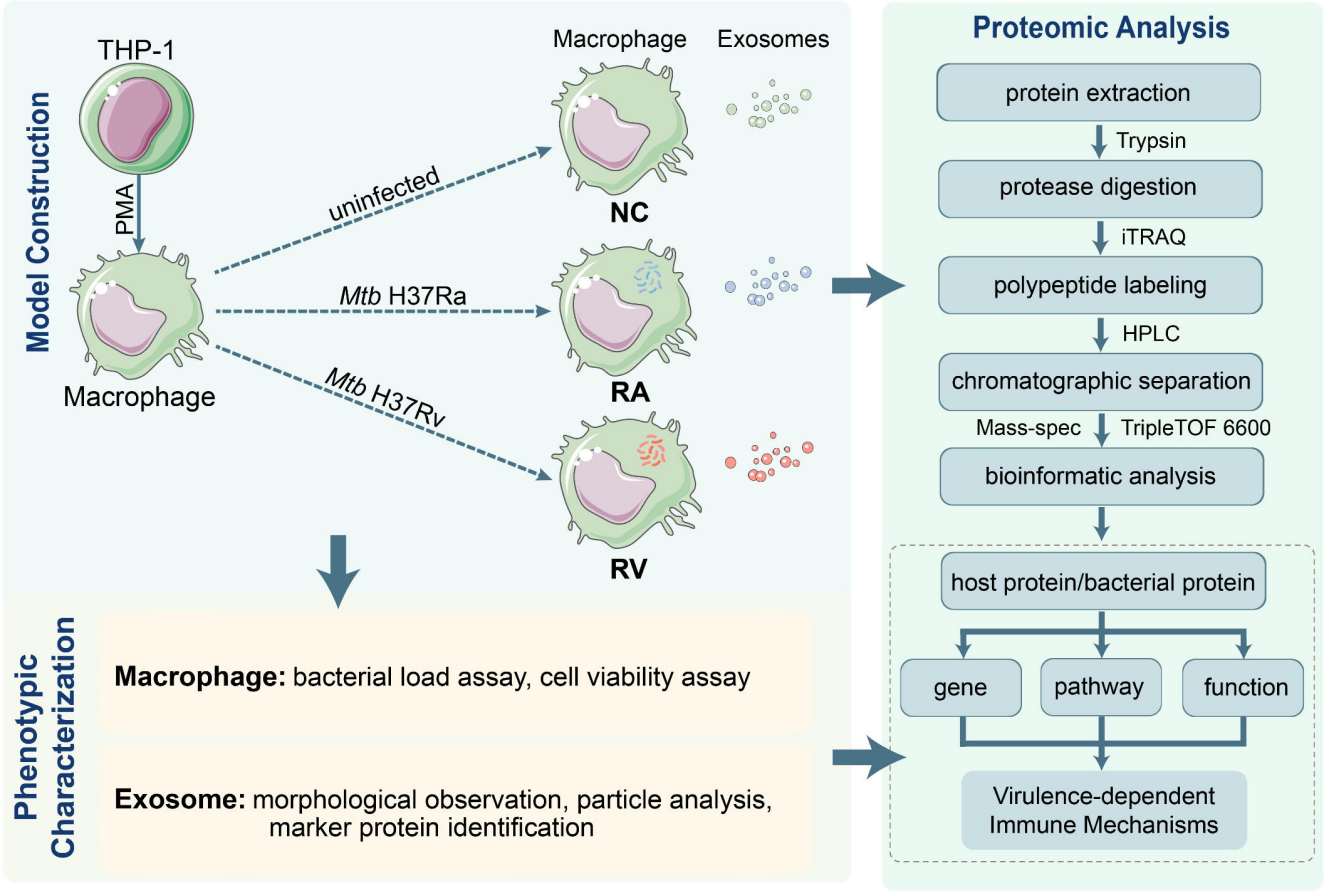
Schematic of the experimental workflow. THP-1 cells differentiate into macrophages (via PMA) and are exposed to Mtb (uninfected/NC, avirulent H37Ra/RA, virulent H37Rv/RV). Macrophages undergo bacterial load/cell viability assays; exosomes undergo morphology/particle/marker protein characterization. Macrophages and released exosomes are analyzed via proteomics (extraction→trypsin digestion→iTRAQ labeling→HPLC separation→TripleTOF 6600 mass spec→bioinformatics). Host/bacterial proteins are analyzed for differentially expressed genes, enriched pathways and functions to explore virulence - dependent immune mechanisms.

**Figure 2:**
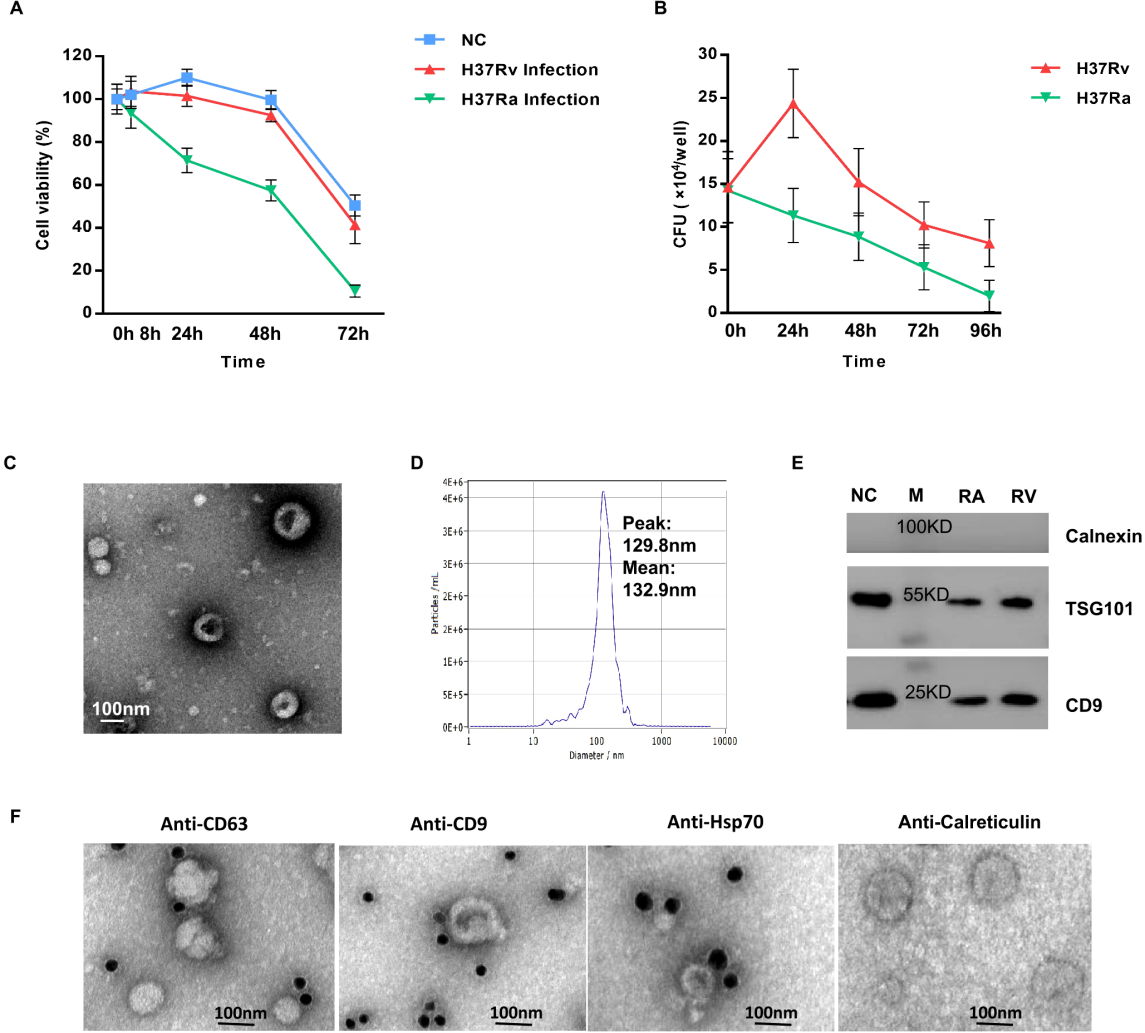
Identification of isolated macrophage-derived exosomes. **(A)** Macrophage viability was assessed via CCK-8 assay at 8, 24, 48, 72 h post-infection, and absorbance read at 450 nm. **(B)** Bacterial survival was measured by CFU assay at 24, 48, 72, 96 h post-infection. Lysates were serially diluted, plated on Middlebrook 7H11 agar (10% OADC), incubated at 37 °C for 3–4 weeks, and colonies counted. **(C)** Transmission electron microscopy (TEM) Image of exosomes (magnification scale bar=100 nm). **(D)** Nanoparticle tracking analysis (NTA) showed the size distribution of isolated exosomes, Y-axis present concentrations (particles/ml), the X-axis presented diameters (nm). The distribution peaks of exosomes are at ~130  nm. **(E)** Western blot examined the exosomes surface marker of TSG101 and CD9, the expression of calnexin (an endoplasmic reticulum marker protein) in vesicles. **(F)** The images of immunogold electron microscopy showing the marker proteins in exosomes (scale bar=100 nm). The exosomal marker proteins of CD9, CD63, and TSG101 were labeled by immunogold particles, with calreticulin (another endoplasmic reticulum marker protein) as negative control.

In this study, *Mtb* virulent strain H37Rv (RV) and avirulent strain H37Ra (RA) were used to infect THP-1 differentiated macrophage, with uninfected cells as the control group (NC) (**Figure 1**). CCK-8 assay revealed distinct viability profiles: H37Rv-infected macrophages showed increased viability during 0-24h post-infection followed by decline (similar to NC), while H37Ra-infected cells exhibited progressive cytotoxicity from initial infection (**Figure 2A**). Consistent with these trends, CFU assays showed H37Rv proliferated intracellularly within 24h before declining, whereas H37Ra loads decreased continuously (**Figure 2B**). Transmission electron microscopy (TEM) and NanoSight Tracking Analysis (NTA) revealed typical cup-shaped exosomes with peak diameters of 129.8 nm (**Figure 2C**) and mean diameters of 132.9 ± 54.3 nm (**Figure 2D**). Western blotting confirmed the presence of exosomal markers CD9 (24 kDa) and TSG101 (44 kDa), without Calnexin (90 kDa) contamination (**Figure 2E**). Besides, CD9, CD63 and Hsp70 could be all detected in the exosomes by the immunogold particles with undetectable Calreticulin (**Figure 2F**).

### 3.2 *Mtb*-infected macrophages and releasing exosomes showed distinct protein expression patterns

**Figure 3:**
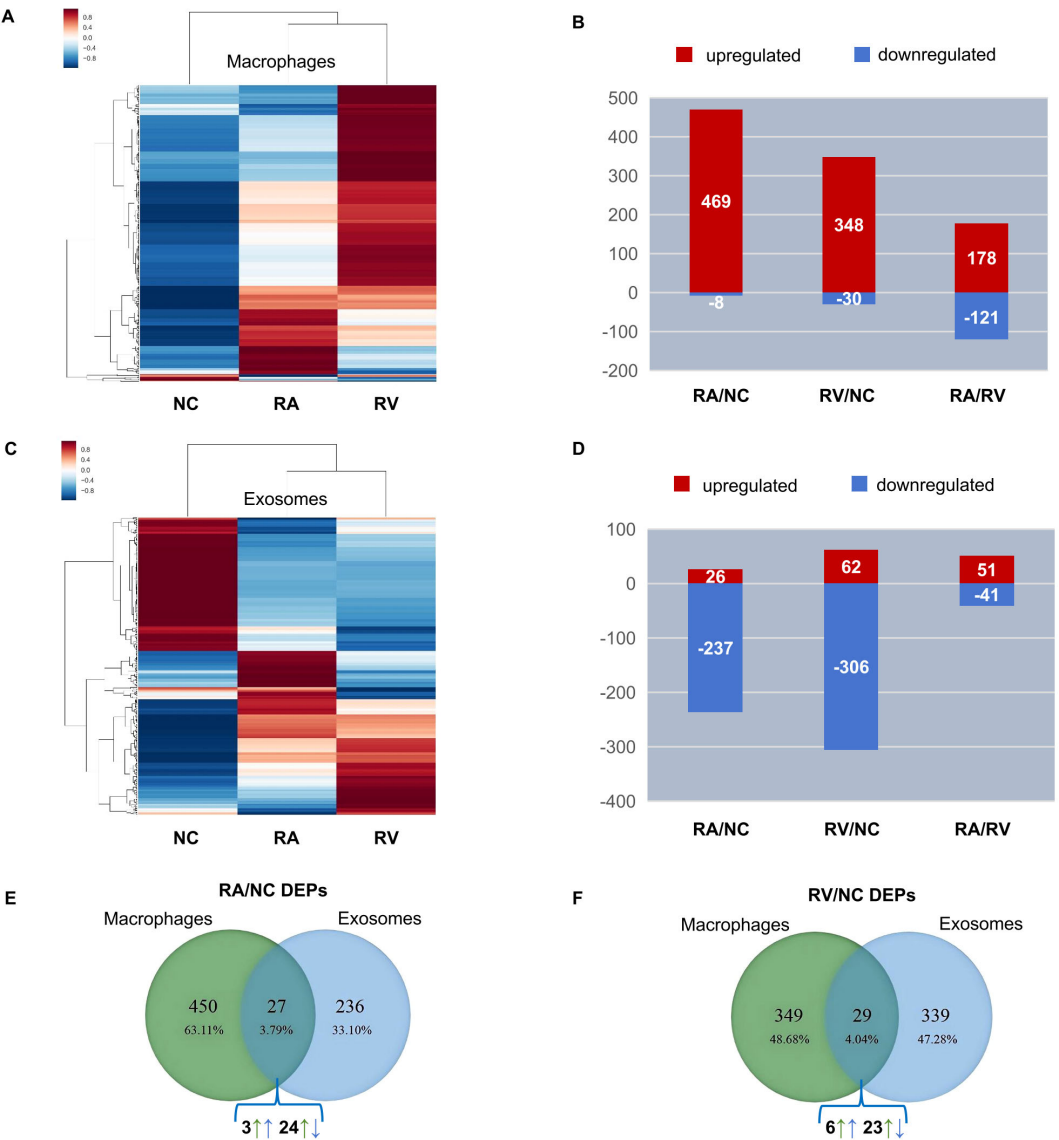
Proteomic profiling of host differentially expressed proteins (DEPs) in macrophages and exosomes. **(A)** Heatmap of DEPs in macrophages showing the expressions of the DEPs in macrophages from three groups (|Fold change| ≥ 1.2, p ≤ 0.05). The color key indicates the expression level of the proteins. **(B)** The bar plots showed the numbers of DEPs in H37Ra (RA) and H37Rv (RV) infected macrophages compared with uninfected NC. Red bars indicated the number of up-regulated proteins in each sample, while the blue bars indicated the number of down-regulated proteins in each sample. **(C)** Heatmap of DEPs in exosomes from three groups (|Fold change| ≥ 1.2, p ≤ 0.05). The color key indicates the expression level of the proteins. **(D)** The bar plots showed the numbers of DEPs in exosomes of RA and RV infected macrophages compared with those of uninfected NC. Red bars indicated the number of up-regulated proteins in each sample, while the blue bars indicated the number of down-regulated proteins in each sample. **(E)** Venn diagram of RA/NC DEPs shared between macrophages and exosomes. Among the shared 27 proteins, 24 (88.9%) showed inverse regulation (up in cells, down in exosomes or vice versa). **(F)** Venn diagram of RV/NC DEPs shared between macrophages and exosomes. Only 29 DEPs overlapped, with 23 (79.3%) being inversely regulated.

ProteinPilot 5.0 software was used for protein searching and data analysis. The basic data and the number of differential proteins in the human proteome are shown in **Table 1**. We identified a large number of human proteins including a total of 3,329 macrophage proteins (3,157 quantifiable) and 983 exosomal proteins (948 quantifiable) respectively. Comparing the similarity between the three groups of differentially expressed proteins (RA/NC, RV/NC and RA/RV), we performed a heat map analysis (**Figure 3A**). The results showed that there was a distinct expression pattern between the NC group and the infected group of *Mtb*, while there was also a significant difference between the RA and RV groups. RA induced more DEPs in macrophages (RA/NC: 477) vs. H37Rv (RV/NC: 378), with 98.3% upregulated in RA vs. 92.1% in RV (**Table 1**, **Figures 3A, B**). Conversely, exosomes showed greater DEPs in RV/NC (368) vs. RA/NC (263) (**Table 1**), with predominant downregulation (RA: 89.8%; RV: 83.1%; **Figures 3C, D**). The hierarchical clustering of DEPs (**Figures 3A, C**) visually reinforces the strain-specific signatures. In macrophages, RV infection induced a more pronounced upregulation profile (warmer red cluster) compared to RV, aligning with its stronger activation of immune pathways. Conversely, the exosomal proteome was dominated by downregulation, with RV-exosomes showing a broader and deeper suppression (cooler blue tones) than RA-exosomes, consistent with its role in systemic immune evasion.

**Table 1:** Basic data of human proteome in macrophages and exosomes.

Items	Macrophages	Exosomes
No. of Total peptides	666,095	720,838
No. of qualitative proteins	3,329	983
No. of quantitative proteins	3,157	948
RA/NC DEPs	477	263
RV/NC DEPs	378	368
RA/RV DEPs	299	92

Comparing DEPs of RA/NC and RV/NC between macrophages and exosomes, we found that minimal overlap existed between cellular/exosomal DEPs that RA/NC shared 27 DEPs (24 inversely regulated) and RV/NC shared 29 DEPs (23 inversely regulated) (**Figures 3E, F**), demonstrating that most DEPs were specifically expressed. Among the 24 RA/NC proteins and 23 RV/NC proteins, seven proteins overlapped across strains, including two proteins located on cell organelle (GUSB, MPST), four mainly in cytosol (GALM, PGAM1, GARS1, GLO1) and one mainly on plasma membrane (ITGB2) (**Table 2**). The specific intracellular retention and exosomal exclusion of these shared proteins suggest a concerted host-pathogen strategy to compartmentalize immune functions. For instance, ITGB2, a key subunit of leukocyte integrins critical for cell adhesion and migration, was significantly upregulated within infected macrophages (RA/NC: 1.32; RV/NC: 1.69) but markedly downregulated in exosomes (RA/NC: 0.16; RV/NC: 0.12). This pattern implies that *Mtb* infection enhances integrin-mediated adhesion and signaling within the host cell to potentially facilitate its intracellular niche, while simultaneously limiting the release of pro-adhesive ITGB2 via exosomes, which could otherwise promote immune cell recruitment and activation at distant sites (25). The inverse regulation of other shared proteins (e.g., GUSB, GLO1) involved in lysosomal function and metabolic stress further supports the notion of bidirectional sorting, where pathways supporting intracellular survival are amplified in the cell, while immunomodulatory or stress-related signals are selectively dampened in the vesicular cargo.

**Table 2:** Seven DEPs of RA/NC and RV/NC shared by macrophages and exosomes.

Accession	Protein	Location	Macrophages		Exosomes	
			RA/NC	RV/NC	RA/NC	RV/NC
P08236	GUSB	Lysosome	1.216	1.458	0.060	0.045
Q96C23	GALM	Cytosol; Exosome	1.346	1.576	0.099	0.077
P05107	ITGB2	Plasma membrane	1.320	1.688	0.158	0.123
P25325	MPST	Mitochondrion	1.220	1.852	0.256	0.314
P18669	PGAM1	Cytosol; Exosome	1.496	2.017	0.503	0.536
P41250	GARS1	Cytosol; Mitochondrion; Exosome	1.313	1.900	0.739	0.454
Q04760	GLO1	Cytosol; Nucleoplasm; Plasma membrane	1.810	2.272	0.818	0.476

### 3.3 IPA analysis reveals bidirectional dysregulation in macrophages and exosomes in response to virulent and attenuated *Mtb*

**Figure 4:**
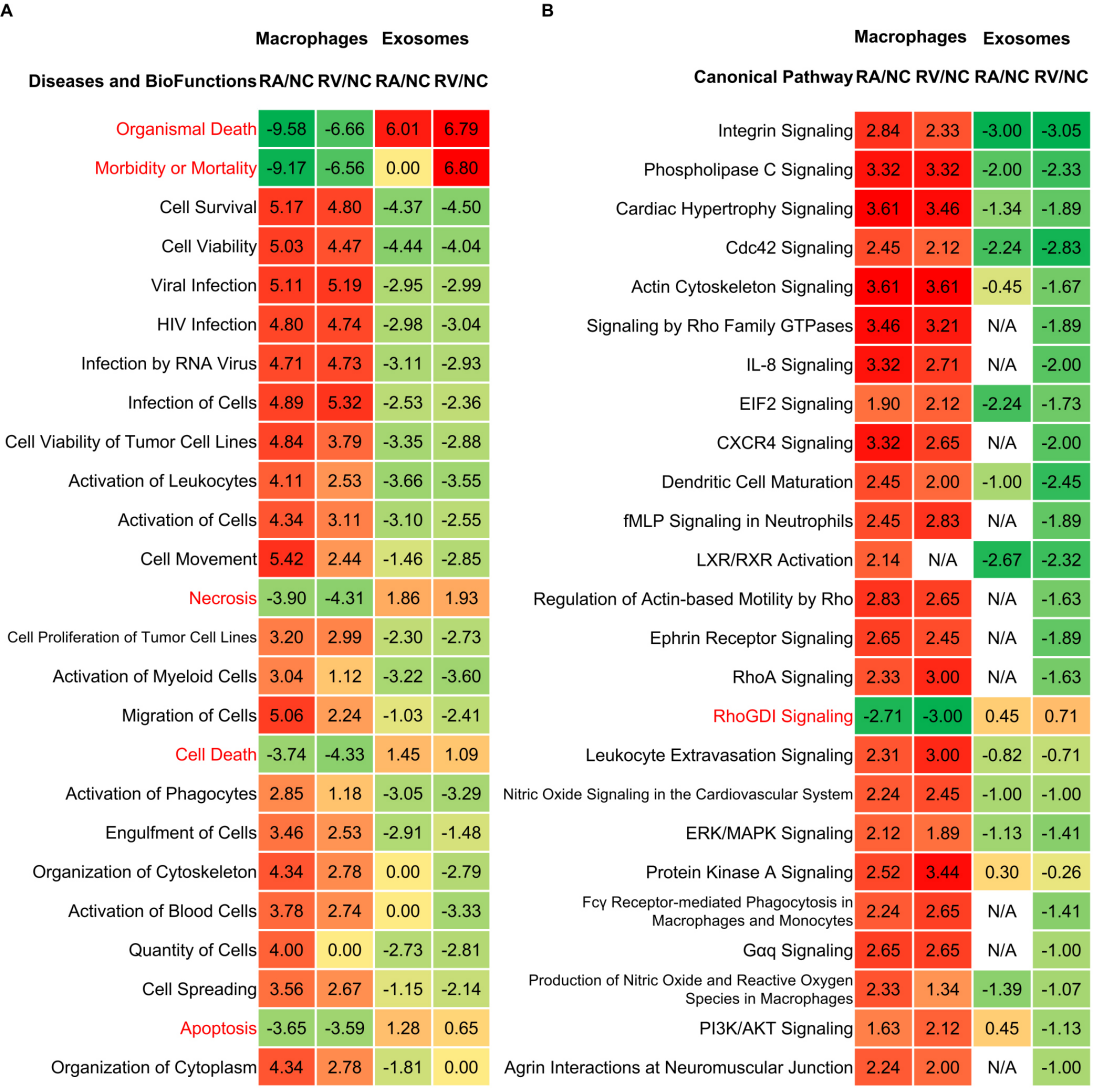
Function and pathway dysregulation between RA/NC and RV/NC in macrophages and exosomes. **(A)** The Ingenuity Pathway Analysis (IPA) Diseases and BioFuctions analysis indicating the Top 25 items. **(B)** The IPA canonical pathway analysis indicating the Top 25 pathways. Z-score represents the IPA regulation trends [Z-score > 0: up-regulation (red); Z-score < 0: down-regulation (green)].

In order to compare the differences in functions and pathways in macrophages and exosomes after *Mtb* infection, we analyzed the DEPs using IPA software and ranked them according to the degree of significance of the differences (z-score). Top 25 IPA functions showed 20/25 upregulated in macrophages but downregulated in exosomes included “Cell Survival”, “Cell Viability”, and “Viral Infection” (**Figure 4A**). Conversely, death-associated functions, such as “Organismal Death”, “Morbidity or Mortality”, “Necrosis”, “Cell Death”, and “Apoptosis”, were downregulated intracellularly but enriched in exosomes (**Figure 4A**). Pathway analysis mirrored this trend: 24/25 pathways, such as “Integrin Signaling”, “Phospholipase C Signaling”, “Cardiac Hypertrophy Signaling”, were upregulated in macrophages but suppressed in exosomes (**Figure 4B**). Critically, “RhoGDI Signaling” was exclusively upregulated in exosomes (z-score >2; **Figure 4B**), implicating its role in cytoskeletal paralysis, phagocytosis, phagosome maturation and inflammatory responses of recipient cells (16). We re-examined our prior clinical exosomal RNA profiling data (17) and found that latent tuberculosis infection (LTBI) upregulated gene expression of *ARHGDIA* (coding RhoGDI) and *CASP9* (coding Caspase-9) compared to healthy controls (HC) and active TB patients (ATB) (**Supplementary Figure S1A**, **Supplementary Table S9**). Followed this with protein-level validation using ELISA on serum-derived exosomes, we confirmed higher levels of RhoGDI and Caspase-9 in LTBI individuals (**Supplementary Figure S1B, C**). This is a direct example of how exosomal cargo may correlate with infection stages.

**Figure 5:**
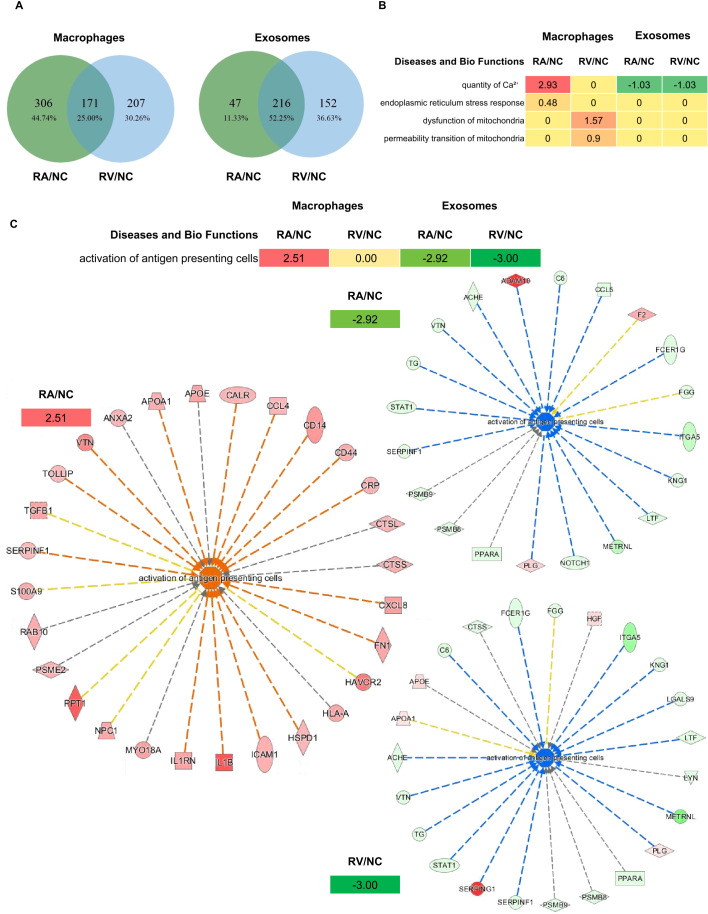
IPA analysis of the overlapped DEPs between RA/NC and RV/NC in macrophages and exosomes. **(A)** Venn diagrams comparing shared responses. In macrophages, RA/NC and RV/NC shared 171 DEPs (25.0% of total DEPs). In exosomes, a greater proportion of DEPs were shared (216, 52.1%). **(B)** One of the main items in Disease and Biofunctions was”activation of antigen presenting cells”. **(C)** Four selected IPA items in Disease and BioFunctions. Z-score represents the IPA regulation trends [Z-score > 0: up-regulation (red); Z-score < 0: down-regulation (green)].

To further examine the distinctions between different virulent strains on macrophages and releasing exosomes, overlapped DEPs of RA/NC and RV/NC were identified and analyzed (**Figure 5**). The data showed that there were fewer DEPs shared by the two compared groups in cells (171, 25.00%, whereas more DEPs were shared by the two groups in exosomal samples (216, 52.05%) (**Figure 5A**). These shared DEPs in macrophages and exosomes were analyzed by IPA platform to examine the changes of functions and pathways after *Mtb* infection. The results showed strain-specific differences: RA/NC macrophages showed robust “antigen presenting cell activation” (z=2.51; **Figure 5B**) and ER stress (z=2.93; **Figure 5C**), while RV/NC induced mitochondrial dysfunction (z=1.57; **Figure 5B**). The Venn diagram analysis (**Figures 3C, F**, **5A**) quantifies the compartmentalization and strain-specificity of the host response. The strikingly low overlap between macrophage and exosomal DEPs, coupled with the high frequency of inverse regulation, strongly supports a model of “bidirectional immune dysregulation”. Furthermore, the higher concordance in exosomal DEPs across strains (**Figure 5A**) implies that manipulating the extracellular milieu via vesicles might be a core, conserved strategy of *Mtb*, even if the intracellular survival tactics differ.

### 3.4 Mycobacterial protein sorting in macrophages and exosomes after *Mtb* infection

**Figure 6:**
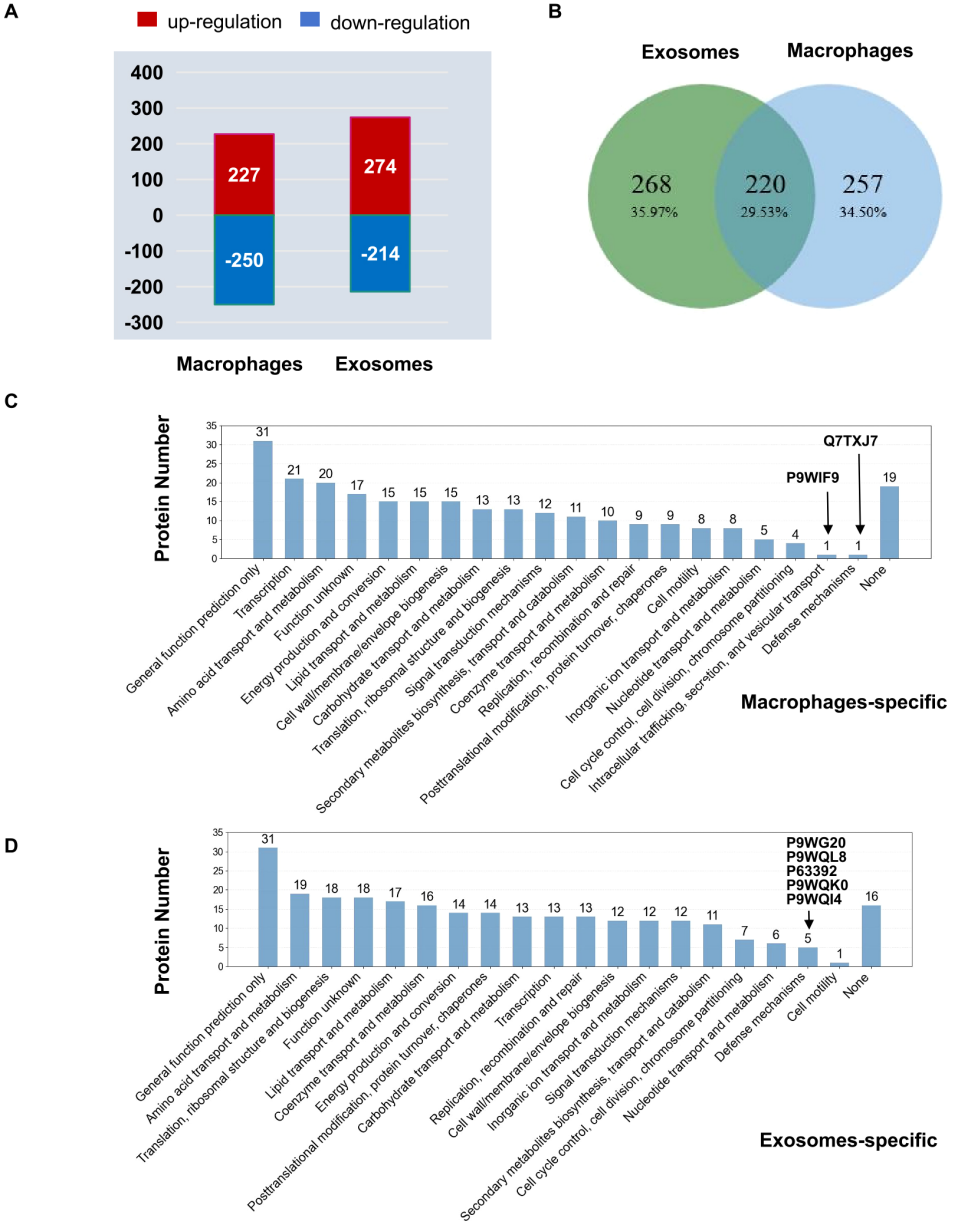
Profiling of differential expressed RA/RV Mtb proteins in macrophages and exosomes. **(A)** The bar plots showed the numbers of DEPs in RA/RV macrophages and exosomes. Red bars indicated the number of up-regulated Mtb proteins, while the blue bars indicated the number of down-regulated Mtb proteins. **(B)** Venn diagram showing the unique and overlapped RA/RV DEPs between the Macrophages and exosomes samples. **(C)** COG clustering of unique RA/RV DEPs in macrophages. **(D)** COG clustering of unique RA/RV DEPs in exosomes. Vertical axis: number of proteins; horizontal axis: category of COG function annotation.

We identified large amounts of *Mtb* proteins in both macrophage and exosome samples, including 2,404 *Mtb* proteins in macrophage and 2,415 *Mtb* proteins in exosmes with 2,100 (87.4%) and 2,129 (88.1%) quantifiable, respectively (**Table 3**). RA-infected exosomes exhibited more DEPs (488) compared to RV-infected macrophages (477) (**Table 3**). By examining the expression trends, macrophages exhibited 52.4% (250/477) of DEPs down-regulated, while exosomes had 56.1% (274/488) of DEPs up-regulated (**Figure 6A**). Then Venn diagram analysis revealed the proportions of shared DEPs and specific DEPs in cells and exosomes: 257 (34.50%) cell-specific DEPs, 268 (35.97%) exosome-specific DEPs and 220 (29.53%) DEPs shared by macrophages and exosome (**Figure 6B**). According to COG functional classification, exosome-specific DEPs were enriched in “translation/ribosomal biogenesis” (18 proteins) and “lipid metabolism” (16 proteins), while macrophage-specific DEPs prioritized “transcription” (21 proteins) and “amino acid metabolism” (20 proteins; **Figures 6C, D**).

**Table 3:** Basic data of *Mtb* proteome in macrophages and exosomes.

Items	Macrophages	Exosomes
Total *Mtb* peptides	922,093	799,118
No. of qualitative *Mtb* proteins	2,404	2,415
No. of quantitative *Mtb* proteins	2,100	2,129
RA/RV *Mtb* DEPs	477	488

Of interest, however, macrophages were annotated to one of the extra categories that is “Intracellular trafficking, secretion, and vesicular transport” (**Figure 6C**). This category of differential proteins was represented by only one P9WIF9 (PE_PGRS20), an antigenic protein of enhancing immune response of host macrophages (18, 19). Its expression was significantly higher in the RA group than in the RV group, with a RA/RV ratio of 1.34 (**Table 4**), suggesting that the avirulent strain *Mtb* could activate intensive immune responses after infection of macrophages. For the “Defense mechanisms” class of unique differential proteins related to immunity, there was only one (Q7TXJ7) in macrophages (**Figure 6C**), which was significantly lower in the RA group than in the RV group (RA/RV=0.549) (**Table 4**), there were five in exosomes including two ABC transporters DrrC (P9WG20; RA/RV=0.624) and DrrA (P9WQL8; RA/RV=0.765), and another three ATP binding proteins P63392 (RA/RV=1.218), P9WQK0 (RA/RV=1.252), and P9WQI4 (RA/RV=2.910) (**Figure 6D**; **Table 4**), indicating strain-specific cargo sorting with immunomodulatory implications.

**Table 4:** *Mtb* strain-specific proteins in the infected macrophages and releasing exosomes.

Samples	Accession	Protein	RA/RV	COG
Macophage	P9WIF9	PE_PGRS20	1.34	Intracellular trafficking, secretion, and vesicular transport
	Q7TXJ7	BQ2027_MB2983C	0.549	Defense mechanisms
Exosomes	P9WG20	DrrC	0.624	Defense mechanisms
	P9WQL8	DrrA	0.765	
	P63392	IrtA	1.218	
	P9WQK0	MT1014	1.252	
	P9WQI4	MT2640	2.910	

### 3.5 Virulence-associated exosomal cargo in immune activation and host-pathogen interaction

In addition we focused on the expression of functional proteins in the RA/RV bacterial DEPs, and the data revealed that functional *Mtb* proteins were preferentially sorted into exosomes: exosomal samples contained more virulence proteins (60 vs. 53), antigenic proteins (65 vs. 63), and membrane-associated proteins (95 vs. 93) than macrophages (**Table 5**).

**Table 5:** The numbers of functional *Mtb* proteins in RA/RV DEPs of macrophages and exosomes.

Samples	Total DEPs	Virulence proteins	Antigenic proteins	Membrane associated proteins
Macrophages	477	53	63	93
Exosomes	488	60	65	95

**Figure 7:**
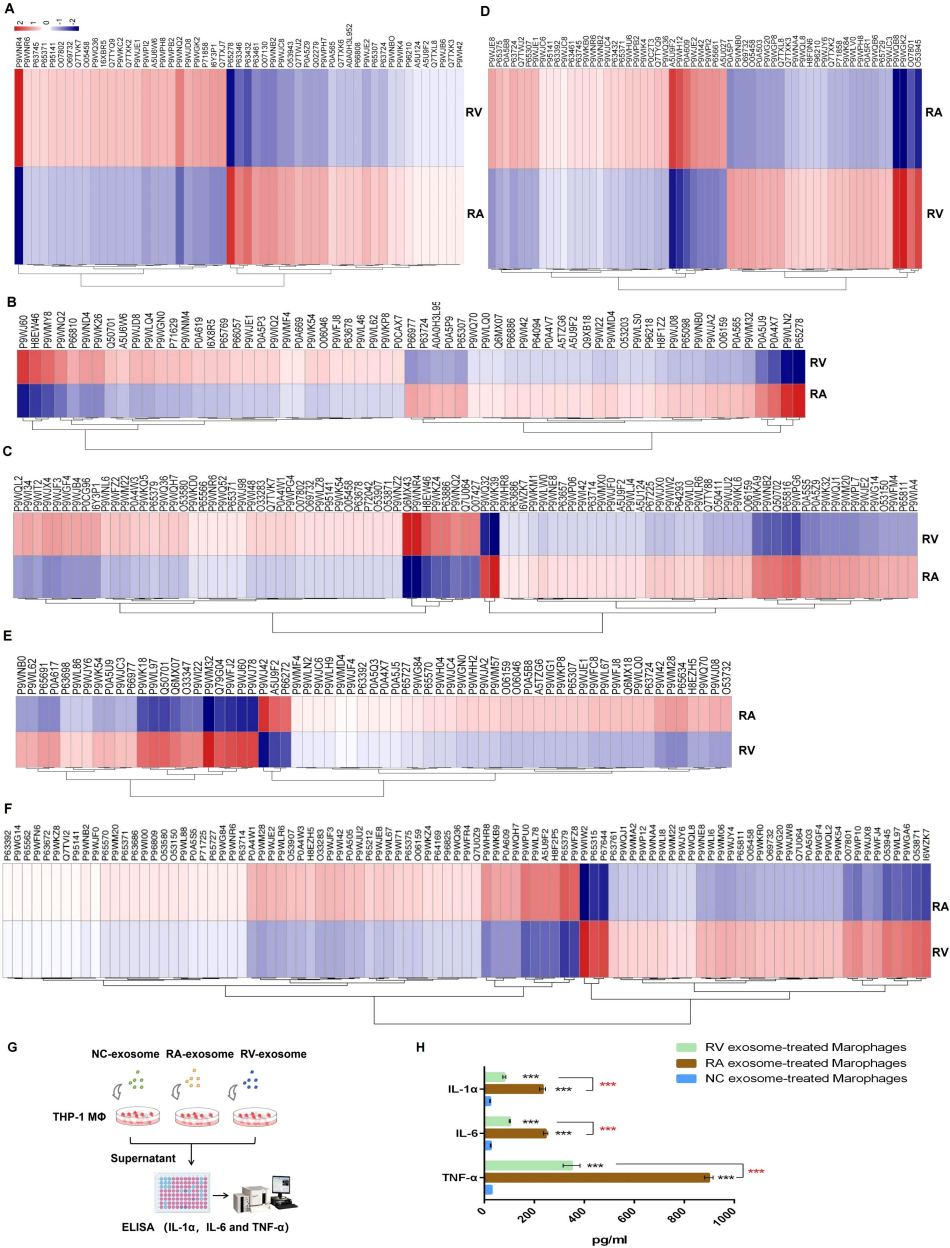
Heat map of functional proteins in RA/RV DEPs. **(A)** Virulence proteins in macrophages. **(B)** Antigenic proteins in macrophages. **(C)** Membrane associated proteins in macrophages. **(D)** Virulence proteins in exosomes. **(E)** Antigenic proteins in exosomes. **(F)** Membrane associated proteins in exosomes. Red bar represents up-regulation and blue bar represents down-regulation. The color key indicates the expression levels of the proteins. **(G)** Workflow of cytokines detection of NC-, RA- RV- exosome treated recipient THP-1 derived macrophages. **(H)** ELISA data showed exosomes from RA-infected donors triggered a significantly stronger pro-inflammatory response in recipient macrophages. Data are mean ± SD and representative of three independent experiments. ***, P < 0.001.

According to the heat map analysis, we discovered some functional proteins with obvious differences between RA/RV in cells (**Figures 7A-C**) and exosome (**Figures 7D-F**). For example, the virulence proteins P9WNR4 (EccB2) and P66947 (ivlG), the antigenic proteins P9WJ60 (MT3488) and P9WLN2 (MT2060) and the membrane-associated protein Q6MX43 (SecE2) were detected in the cellular samples. While the virulence proteins A5U9F2 (MmpL8), P9WGK2 (DevS) and O53945 (MycP5), the antigenic protein P9WF42 (WhiB1) and P45813 (RpsM), and the membrane-associated proteins P65315 (LprF), P9WFZ8 (OppC), P65379 (MmpS3), and P9WPU0 (CtpA) were identified in the exosomal samples (**Table 6**). Notably, RA-exosomes showed upregulation of 5/8 key proteins (RA/RV>3), while RA-macrophages downregulated 3/4 (RA/RV<1), indicating avirulent strains export functional proteins via exosomes.

**Table 6:** Some functional *Mtb* proteins in RA/RV DEPs of macrophages and exosomes.

Sample	Accession	Protein	RA/RV	Sample	Accession	Protein	RA/RV
Macrophages	P9WNR4	EccB2	0.173	Exosomes	A5U9F2	MmpL8	3.672
Macrophages	P9WJ60	MT3488	0.231	Exosomes	P65379	MmpS3	6.243
Macrophages	P66947	IlvG	0.322	Exosomes	P9WFZ8	OppC	4.882
Macrophages	Q6MX43	SecE2	0.161	Exosomes	P9WF42	WhiB1	6.19
Exosomes	P9WGK2	DevS	0.233	Exosomes	P9WPU0	CtpA	4.122
Exosomes	P65315	LprF	0.199	Exosomes	P45813	RpsM	100
Exosomes	O53945	MycP5	0.323	Macrophages	P9WLN2	MT2060	4.293

Specifically, We performed an *in vitro* co-culture assay where recipient THP-1-derived macrophages were treated with exosomes isolated from RA- or RV-infected macrophages. Exosomes from RA-infected donors triggered a significantly stronger pro-inflammatory response in recipient cells, marked by elevated secretion of TNF-α, IL-1α, and IL-6, compared to those from RV or NC (**Figures 7G, H**), which directly validated the immune-activating role of H37Ra exosomes suggested by our proteomic data.

## 4 Discussion

**Figure 8:**
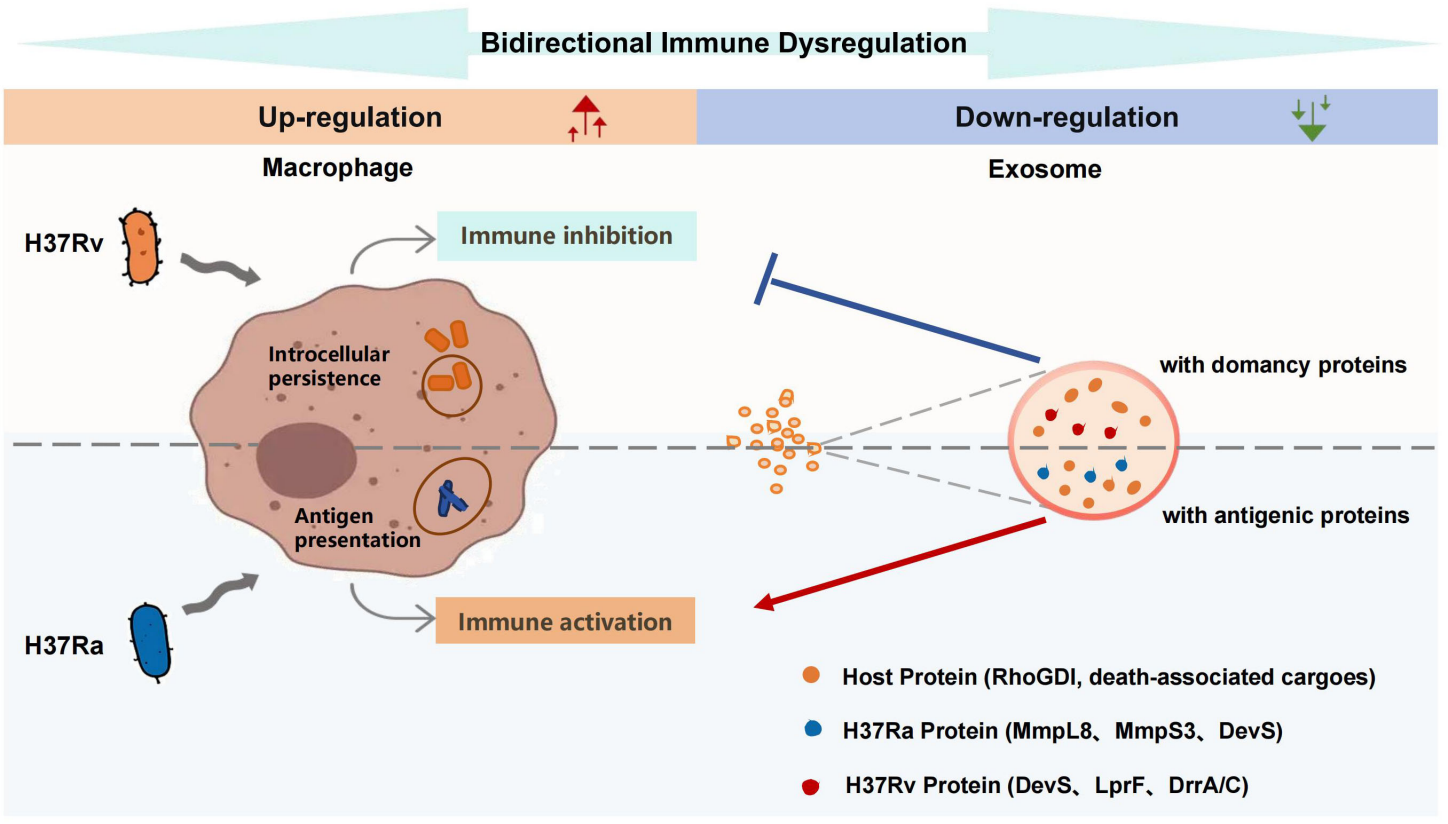
Proposed model of exosome-mediated bidirectional immune dysregulation by *Mtb* strains H37Rv and H37Ra. This schematic summarizes the strain-specific strategies through which *Mtb* modulates host immunity via exosomal communication.

This study delineates how *Mtb* subverts macrophage function and exploits exosomal trafficking to orchestrate immune evasion, revealing virulence-dependent strategies through comparative proteomic and functional analyses of virulent H37Rv (RV) and attenuated H37Ra (RA) strains (**Figure 8**).

### 4.1 Virulence dictates host cell fate and bacterial survival

We found that RV-infected macrophages maintained viability similar to controls, suggesting successful immune evasion through latency induction, which aligns with previous reports of RV’s necrotic phenotype (5, 6). The significantly higher survival rate of RV may be attributed to its enhanced expression of invasion-associated proteins such as MmpL8 (A5U9F2), which mediates trehalose dimycolate transport and phagosomal membrane remodeling (20, 21). And moreover, RV downregulated the ESX-2 secretion system component EccB2 (P9WNR4) by 5.8-fold in macrophages (**Table 6**), impairing effector delivery and cytotoxicity (22, 23). Conversely, RA infection triggered apoptotic cell death, supported by continuous viability decline (~90% at 72 h) and upregulation of pro-apoptotic antigens like PE_PGRS20 (P9WIF9) in macrophage (**Figure 2A**, **6C**; **Table 5**) (18, 19, 24). Furthermore, RA triggers ER stress and calcium dysregulation (z=2.93), while RV targets mitochondria (dysfunction z=1.57; permeability z=0.90) (**Figure 5C**), highlighting strain-specific organelle sabotage for survival.

### 4.2 Exosomes as “virulence vectors” for immune subversion

Strikingly, bidirectional pathway dysregulation was observed: 24/25 top IPA pathways were upregulated intracellularly but downregulated in exosomes (**Figure 4**). For example, infected macrophages upregulate pro-survival pathways (Integrin/Phospholipase C/Cardiac Hypertrophy Signaling) (25–28) but selectively export death-inducing cargo (e.g., Caspase-9) and RhoGDI (suppression of cytoskeletal dynamics, phagosome maturation, and antigen presentation in recipient cells) (16) into exosomes derived from LTBI (**Supplementary Figure S1**; **Supplementary Table S9**), suggesting that *Mtb* may hijack host sorting machinery to retain pro-survival signals intracellularly while packaging immunosuppressive or death-modulating signals into exosomes to manipulate the systemic environment. This “bidirectional sorting” may be a strategy for maintaining dormancy and immune paralysis in the host.

### 4.3 Strain-specific cargo sorting and immune modulation

Exosomal analysis revealed strain-specific cargo sorting with immunomodulatory implications. RV exosomes were enriched in dormancy-associated regulators like DevS (P9WGK2; 4.3-fold), a hypoxia adaptation factor (29, 30), and immunosuppressive effectors such as the lipoglycan transporter LprF (P65315; 5.0-fold), which enhances cell wall evasion (31). Conversely, RA exosomes prioritized immunogenic cargoes, which stimulated higher levels of pro-inflammatory cytokines in recipient macrophages (**Figure 7G-H**) including membrane protein MmpS3 (P65379; 6.2-fold), which drives biogenesis of mycobacterial cell envelope (32), and lipid transporter MmpL8 (A5U9F2; 3.7-fold), a phagosomal remodeler critical for trehalose dimycolate transport (20, 21). Human proteome profiling further illuminated RA induced 26.2% more DEPs in macrophages, robustly activating antigen presentation (**Figure 5C**), whereas RV suppressed these pathways to promote latency (33). Shared DEPs between RA/NC and RV/NC reinforced this trend: 24/27 RA/NC and 23/29 RV/NC proteins were upregulated in macrophages but excluded from exosomes, including defense-related Q7TXJ7 which was significantly higher in RV group (1.8-fold), implying a conserved but distinct mechanism to restrict immune activation. Besides, Mtb proteome analysis uncovered that RV-infected macrophages enriched “amino acid transport and metabolism” COG function and upregulated amino acid biosynthesis protein ilvG (P66947; 3.1-fold), supporting intracellular survival (34, 35), while RA exosomes enriched “lipid transport and metabolism” COG function and INH-hypersensitive protein rpsM (P45813; 100-fold), indicative of enhanced antigen processing (36). Notably, RV exosomes prioritized antibiotic resistance mechanisms, upregulating ABC transporters DrrC (P9WG20; 1.6-fold) and DrrA (P9WQL8; 1.3-fold) (37, 38), whereas RA exported secretion-disrupting effectors like secE2 (Q6MX43; 6.2-fold) in cells, impairing phagosome maturation (39, 40). These dichotomy reflects distinct immune regulation strategies: RV suppresses extracellular immune crosstalk via exosomal dormancy factors, while RA leverages exosomes to amplify antigenic signals, despite its attenuated virulence.

### 4.4 Limitations and future directions

While this study provides a comprehensive proteomic landscape of strain-specific exosomal cargo and host responses in *Mtb* infection, several limitations should be acknowledged. First, our mechanistic insights, particularly regarding the role of exosomal RhoGDI signaling in recipient cell paralysis and the functional impact of delivered virulence-associated proteins, are derived from correlative proteomic data and limited clinical verification. Future studies employing functional validation—such as using RhoGDI inhibitors in recipient macrophages or assessing the immunomodulatory effects of isolated exosomes in co-culture systems—are essential to confirm these proposed pathways. Second, the reliance on THP-1-derived macrophages may not fully recapitulate the complexity of the human immune microenvironment. Translational relevance of our findings, including the diagnostic potential of exosomal biomarkers (e.g., RhoGDI, virulence-associated proteins), necessitates validation in more physiologically relevant systems, such as primary human monocyte-derived macrophages, *in vivo* animal models of tuberculosis and larger cohorts. Finally, the molecular mechanisms governing the differential sorting of bacterial proteins into exosomes remain speculative. The potential roles of host machinery (e.g., ESCRT complexes, tetraspanin microdomains) and bacterial secretory systems (e.g., ESX) in directing cargo loading represent critical and testable hypotheses for future research.

## 5 Conclusion

This comprehensive proteomic analysis reveals distinct immune modulation strategies employed by virulent H37Rv and attenuated H37Ra *Mtb* strains, mediated through both macrophage reprogramming and exosomal cargo sorting. H37Rv prioritizes immune evasion by favoring dormancy-associated regulators and antibiotic resistance machinery in exosomes. In contrast, H37Ra triggers apoptotic cell death and amplifies immunogenic signals via exosomal antigens and robust activation of antigen presentation pathways, despite its attenuated phenotype. The bidirectional dysregulation of immune pathways - upregulated intracellularly but suppressed in exosomes - suggests a conserved mechanism for *Mtb* to restrict extracellular immune crosstalk. Notably, strain-specific exosomal biomarkers and virulence-associated proteins identified here offer promising therapeutic targets to disrupt bacterial persistence or enhance host immunity. However, the reliance on *in vitro* models underscores the need for future studies integrating primary human macrophages, animal models, and multicenter clinical validations to translate these findings into actionable diagnostics or host-directed therapies.

## Data Availability

The mass spectrometry proteomics data have been deposited to the ProteomeXchange Consortium (https://proteomecentral.proteomexchange.org) with the dataset identifier PXD072930.
