# Supplementary material for: Exosome-mediated bidirectional immune dysregulation in tuberculosis: proteomic profiling reveals strain-specific strategies of virulent H37Rv and attenuated H37Ra
Source: Front Immunol. 2026 Jan 23;16:1696299. doi: 10.3389/fimmu.2025.1696299 (PMC12876188; doi:10.3389/fimmu.2025.1696299)

**Supplemental Materials**

**Methods:**

1. RNA sequence data re-analysis: Based on our clinical exosomal RNA profiling data (GEO; accession number: GSE94907), we examined gene expression levels of *RHOGDI* and death-related genes among the Differential expressed genes (DEGs, fold change > 2 and p-value < 0.05).
2. Sample collection: Declaration of Helsinki, and the protocol was approved by the Ethics Committee of the Beijing Chest Hospital, Capital Medical University (number of ethical approvals: BJXK-2017-40-01). All of the participants were HIV-negative adults (≥18 years old) and had no TB history before. The LTBI patients were identified based on positive tuberculin skin test (TST) and interferon-gamma release assay (IGRA) results, but their other indicators were the same as healthy controls (HC). For HC individuals, they were identified by normal computed tomography (CT) chest films, negative TST and IGRA results.
3. ELISA: Serum exosomes from above HC, LTBI were isolated by using exo-Easy Maxi Kit according to the manufacturer’s instructions (QIAGEN, Cat.76064). Human RhoGDI and CASP9 were detected using RhoGDI ELISA kit (Antibodies, A5052) and Caspase-9 ELISA kit (Abcam, ab119508) , and analyzed using Luminex 200 platform.

**Figure S1 Validation of exosomal RhoGDI and Caspse 9 expression in clinical HC, LTBI and ATB Samples.** (A) DEGs in exosomal RNA profiling (prior clinical exosome research data) of individuals with latent TB infection (LTBI) revealed upregulation (Based on FPKM value) of *ARHGDIA* (coding RhoGDI) and *CASP9* (coding Caspase-9) compared to healthy controls (HC) and active TB patients (ATB). (B) and (C) ELISA on serum-derived exosomes from LTBI individuals showed significantly higher levels of exosomal RhoGDI and Caspase-9 in LTBI versus HC. All the data are mean±SD and representative of three independent experiments. *, P < 0.05; **, P < 0.01; ***, P < 0.001.


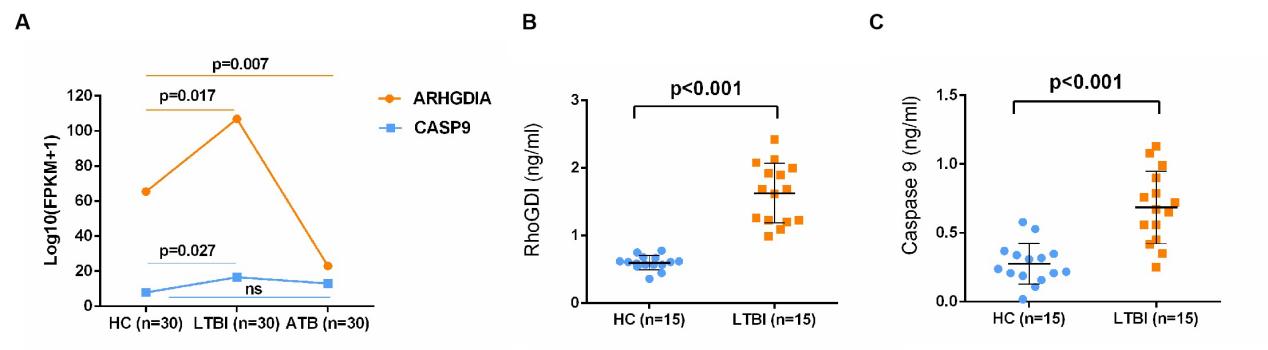

Supplement: Supplementary file 1 [file Supplementaryfile1.docx]
